# *Act1* out of Action: Identifying Reliable Reference Genes in *Trichoderma reesei* for Gene Expression Analysis

**DOI:** 10.3390/jof11050396

**Published:** 2025-05-21

**Authors:** Caroline Danner, Yuriy Karpenko, Robert L. Mach, Astrid R. Mach-Aigner

**Affiliations:** Institute of Chemical, Environmental and Bioscience Engineering, Technische Universität Wien, Gumpendorfer Str. 1a, 1060 Vienna, Austria; caroline.danner@tuwien.ac.at (C.D.); yuriy.karpenko@tuwien.ac.at (Y.K.); robert.mach@tuwien.ac.at (R.L.M.)

**Keywords:** reference gene, actin, gene expression analysis, RT-qPCR, *Trichoderma reesei*

## Abstract

*Trichoderma reesei* is a well-established industrial enzyme producer and has been the subject of extensive research for various applications. The basis of many research studies is the analysis of gene expression, specifically with RT-qPCR, which requires stable reference genes for normalization to yield reliable results. Yet the commonly used reference genes, *act1* and *sar1*, were initially chosen based on reports from the literature rather than systematic validation, raising concerns about their stability. Thus, properly evaluated reference genes for *T. reesei* are lacking. In this study, five potentially new reference genes were identified by analyzing publicly available transcriptome datasets of the *T. reesei* strains QM6a and Rut-C30. Their expression stability was then evaluated under relevant cultivation conditions using RT-qPCR and analyzed with RefFinder. The two most stable candidate reference genes were further validated by normalizing the expression of the well-characterized gene *cbh1* and comparing the results to those obtained using *act1* and *sar1*. Additionally, *act1* and *sar1* were normalized against the new reference genes to assess the variability in their expression. All five new reference genes exhibited a more stable expression than *act1* and *sar1*. Both in silico and RT-qPCR analysis ranked the so far uncharacterized gene, *bzp1*, as the most stable. Further, we found that *act1* and *sar1* have strain- and condition-dependent expression variability, suggesting that they are unsuitable as universal reference genes in *T. reesei*. Based on these results, we propose to use the combination of *bzp1* and *tpc1* for the normalization in RT-qPCR analysis instead of *act1* and *sar1*.

## 1. Introduction

*Trichoderma reesei* is of high industrial and scientific relevance due to its ability to produce and secrete enzymes, such as cellulases and hemicellulases, in large quantities [1]. These enzymes convert lignocellulose into mono- or oligomeric sugars, which are valuable for bioethanol production and are needed in the paper and textile industries and the food sector [2]. To maximize their industrial potential, optimizing enzyme production and bioprocess efficiency is important. Gene expression analysis plays a key role in understanding the regulation of enzyme expression in *T. reesei* and enables targeted strain engineering for enhanced production [3].

Gene expression analysis studies the transcript levels of specific genes or entire transcriptomes to identify differences between conditions or organisms. The basic principle behind different methods is to detect and quantify RNA either directly or indirectly [4,5]. Traditional methods like Northern blot analysis and RNase protection assays have been replaced by more recent techniques such as DNA microarrays, quantitative PCR (qPCR), and whole transcriptome sequencing (WTS) [6,7,8]. While microarrays enable high-throughput analysis, WTS has revolutionized the field by providing a comprehensive, high-resolution view of transcriptome-wide gene expression. Combined with bioinformatics, the later method facilitates the identification of differentially expressed genes, novel transcripts, and key regulatory networks [9].

Among these techniques, reverse transcription–quantitative PCR (RT-qPCR) has become a widely used method due to its high sensitivity, specificity, and cost-effectiveness. RT-qPCR enables the quantification of gene expression by converting RNA into complementary DNA (cDNA) before amplification [10,11]. Reliable reference genes are essential for accurate gene expression quantification [12]. The minimum information for publication of quantitative real-time PCR experiments (MIQE) guidelines emphasizes the importance of selecting reference genes with stable expression across various conditions and relatively high expression and expression levels comparable to the target genes [13]. Computational tools like Genorm [14], Normfinder [15], and BestKeeper [16] or the comparative ΔCt-method [17] help assess gene stability, while WTS has enabled the discovery of novel reference genes [18].

Initially, reference genes for RT-qPCR were selected based on the assumption that their expression remains stable under all experimental conditions [19]. Therefore, housekeeping genes, such as those coding for actin or GAPDH, and ribosomal RNAs are commonly used, as they are involved in essential cellular functions [20]. Often, these genes were not thoroughly characterized in terms of their expression stability. In *T. reesei*, a study in 2010 identified *sar1* as the most stable reference gene among a limited set of candidates that were reported to be stable in other filamentous fungi [21]. *Act1* and *sar1* have since then been commonly used as reference genes in *T. reesei*, despite a limited understanding of their regulation. Recently, studies on filamentous fungi and yeasts have demonstrated that some classical housekeeping genes fail to meet the stability requirements for reference genes, and new, more suitable candidates were discovered. For example, Tao et al. found that traditional reference genes were unsuitable for *Volvariella volvacea* and identified better-performing alternatives [22]. Similar studies on *Amylostereum areolatum* [23] and *Komagataella phaffii* (*Pichia pastoris*) [24] also identified new reference genes, suggesting that more appropriate candidates for *T. reesei* may still be discovered.

In this study, we aimed to identify alternative reference genes for *T. reesei* and evaluated the stability of *act1* and *sar1*. Using publicly available WTS datasets from the *T. reesei* strains QM6a and Rut-C30 under various growth conditions, we identified the most stably expressed genes based on their coefficient of variation (CV), expression levels, and biological function. Five potential reference genes were selected and validated for RT-qPCR using RefFinder [25] with biological samples from QM6a and Rut-C30 cultivated under different conditions, including osmotic and endoplasmic reticulum (ER) stress. The application of the two most promising candidates as reference genes was shown for RT-qPCR in *T. reesei* by normalizing *cbh1* expression. In addition, we provide the first evidence that *act1* and *sar1*, despite their common use, are not universally stable reference genes. Finally, we compared their performance as reference genes with the newly identified candidates under cultivation conditions relevant to cellulase expression and assessed their own expression by normalization using the new reference genes.

## 2. Materials and Methods

### 2.1. Fungal Strains

The following *T. reesei* strains were used for this study: the wild-type strain QM6a (ATCC 13631) and the strain Rut-C30 (ATCC 56765) [26], which is described as hypercellulytic and was derived by two rounds of random mutagenesis and screening from QM6a. The strains were maintained on potato dextrose agar plates. For short-term storage, the strains were kept on plates at 4 °C and, for long-term storage, as spore suspensions in 25% glycerol at −80 °C.

### 2.2. Transcriptome Dataset Processing and Analysis

Publicly available WTS datasets of *T. reesei* strains QM6a and Rut-C30 were retrieved from the EBI FTB database of NCBI [27]. Table A1 and Table A2 in the Appendix A provide an overview of the experimental conditions and technical specifications of these datasets.

The raw reads were processed to obtain raw counts using HISAT2 (v2.2.1) [28], SAMtools (v1.16.1) [29], and featureCounts (v2.0.3) [30,31] in the UNIX-based Debian 12 operating system. The DeSeq2 (v1.14.0) [32] package was used to normalize the raw counts in RStudio (v2024-04-2 Build 764) and then exported as Excel files.

The required genomes and respective annotations QM6a (GenBank GCA_000167675.2) and Rut-C30 (GenBank GCA_000513815.1) were retrieved from GenBank. To enable a direct comparison of gene expression between QM6a and Rut-C30 despite their different annotation, a list of orthologous genes was created in Debian using gffread (v0.12.7) [33] and BLAST (v2.15.0) [34], both installed via the Bioconda package manager (v3.3.1). An exemplary script that was used for all those steps can be found in Appendix B.

Finally, to compare gene stability, the CV was calculated for each gene present in the datasets. Then, the candidate housekeeping genes were selected based on a low CV and a high expression as described in the MIQE guidelines [13].

### 2.3. Cultivation Conditions

To test the differential expression of the new candidate reference genes in common lab experimental conditions, different cultivation conditions typically used for *T. reesei* were tested. This included the use of various carbon sources, cultivation scales, cultivation times, and two common stress conditions. A total of 10^9^ spores per liter (final concentration) were used to inoculate biological duplicates of each condition.

For direct cultivations, the strains were cultivated in 250 mL or 1 L shake flasks at 30 °C and 180 rpm in 50 mL or 200 mL Mandels–Andreotti (MA) medium supplemented with 1% carbon source (glucose, lactose, xylan, glycerin, and cellulose) for 24 to 120 h depending on the carbon source.

To simulate two common occurring stress conditions, osmotic stress, and ER stress, the strains were cultured as described above in MA medium with 1% glucose or lactose, respectively. To induce osmotic stress, concentrated sodium chloride (NaCl) solution was added after 48 h to a final concentration of 1 M. To mimic ER stress, dithiothreitol (DTT) was added after 72 h to a final concentration of 20 mM.

Samples were harvested at two time points depending on the used strain and growth condition to reflect both early and late growth stages. An overview of all growth conditions and harvesting time points can be found in Table 1.

Sample volumes ranged from 2 to 12 mL, depending on the growth stage and mycelium density. Mycelia were obtained by filtration with a textile filter (Miracloth, Calbiochem, San Diego, CA, USA), washed with ultra-pure sterile water, and shock-frozen and stored in liquid nitrogen until RNA extraction.

### 2.4. RNA Extraction

RNA was extracted from approximately 100 mg of frozen mycelium placed in screw-cap tubes containing 1 mL RNAzol, along with 0.37 g small glass beads (0.1 mm diameter), 0.25 g medium glass beads (1 mm diameter), and one large glass bead (5 mm diameter). The mycelia were homogenized using a FastPrep^®^-24 (MP Biomedicals, Santa Ana, CA, USA) at intensity level 6 for 30 s. After homogenization, the tubes were incubated at room temperature for 5 min, then centrifuged at 12,000× *g* for 5 min. RNA was eluted with 30 µL RNase-free water, and its concentration and purity were assessed using a NanoDrop OneC spectrophotometer (Thermo Fisher Scientific, Waltham, MA, USA). RNA purification was performed using the RNA extraction kit from Zymo Research (Zymo Research, Tustin, CA, USA), following the manufacturer’s protocol.

### 2.5. cDNA Synthesis

RNA samples containing 500 ng total RNA were transcribed to cDNA using the LunaScript^®^ RT SuperMix kit (New England Biolabs, Ipswich, MA, USA) on a T100 Thermal Cycler (Bio-Rad Laboratories, Hercules, CA, USA) according to the manufacturer’s guidelines. The resulting cDNA was diluted 1:50 with ultrapure water for direct usage in qPCR or storage at −20 °C.

### 2.6. qPCR

The stability of the candidate reference genes was compared to the commonly used housekeeping genes *act1* and *sar1* by qPCR. For this, the Luna^®^ Universal (RT)-qPCR kit (New England Biolabs, Ipswich, MA, USA) was used following the manufacturer’s guidelines. Each reaction consisted of 2 µL of diluted cDNA as a template and 13 µL of master mix, and measurements were performed in technical duplicates. A QIAgility pipetting robot (QIAGEN, Hilden, Germany) was used for the automated preparation of the qPCR reaction mixes. For every primer pair a no-template control was prepared. The standard Luna^®^ qPCR cycle was performed using a Rotor-Gene Q system (QIAGEN, Hilden, Germany) with version 2.3.1 software. The primer sequences can be found in Table 2. Primer specificity was assessed by melting curve analysis. Additionally, the qPCR product size was verified on an agarose gel and confirmed by sequencing. PCR efficiencies were calculated through the Rotor-Gene software for every run and all were above 1.74.

### 2.7. Data Analysis

To evaluate gene stability, the resulting Ct values from the qPCR were processed with RefFinder [25]. This online tool combines four computational methods, the comparative ΔCt-method [17], BestKeeper [16], Normfinder [15], and Genorm [14], to calculate a final ranking that allows to compare gene expression stability. The individual rankings and calculated values that led to the final composite score of RefFinder are reported in the Appendix B in Table A4. In addition to a combined result of all cultivation conditions, we formed different data subsets to assess eventual gene stability differences depending on the used strains or early and late time points.

## 3. Results

### 3.1. Identification of New Candidate Reference Genes for T. reesei Using WTS Data

**Figure 1:**
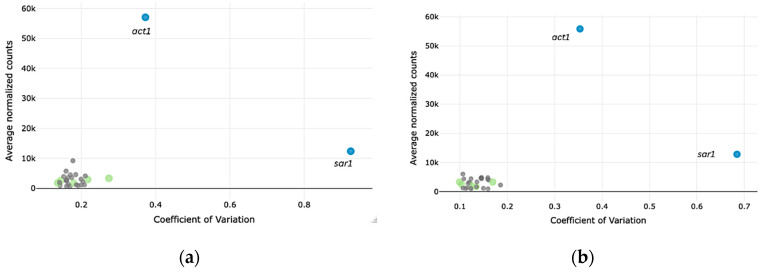
Average normalized counts plotted against the CV of the top 25 ranked stable genes found in transcriptome datasets in comparison to *act1* and *sar1* for the *T. reesei* strains QM6a (**a**) and Rut-C30 (**b**). Based on the metadata provided with the publicly available WTS datasets, biological duplicates were included for each cultivation condition. Blue dots, *sar1* and *act1*; green dots, the candidate reference genes that were analyzed further; gray dots, the remaining 20 stable genes.

In the past, reference genes were selected for validation mainly based on reports from the literature. Nowadays, the availability of WTS data allowed us to select new candidate reference genes based on the MIQE guidelines [13] through bioinformatic analysis of such available WTS datasets for the *T. reesei* strains QM6a and Rut-C30. Potentially suitable reference genes were filtered for a low CV and a medium to high average expression. This approach already revealed many genes that appeared more stable than the commonly used genes for normalization of qPCR data, *act1* and *sar1*. Figure 1 shows the CV plotted against the average expression of the 25 most stable ranked genes and of *act1* and *sar1*. All of the 25 have a much lower CV than *act1* and *sar1*.

Based on these results and in consideration of the function of those genes, five candidate genes were chosen for further validation using RT-qPCR. The gene *bzp1* was annotated in NCBI as a protein of unknown function. Through eggnog [35], gene orthologs were identified in multiple filamentous fungi from the subdivision pezizomycotina (a division of Ascomycota), and over String-db.org [36] and Uniprot, the protein structure and clusters of interacting proteins were analyzed. The protein structure prediction resulted in a bZIP domain. An orthologue in *Fusarium oxysporum f. sp. cubense* was assigned the name “bZIP domain-containing protein” by Guo L. et al. [37]. Thus, this name was adopted for the gene of *T. reesei*. The bZIP domain has DNA binding properties and proteins with this domain are usually transcription factors. In some filamentous fungi, bZIP transcription factors are related to the regulation of stress responses [38,39]. Connections to histone H4, several chromatin remodeling proteins, and arginine methyltransferase were found. Therefore, it could be hypothesized that it is a factor in the process of histone methylation.

The gene *tpc1* includes a Gryzun domain, which is reported to be responsible for protein trafficking through membranes [40]. Both *cue1* and *ubi1* are part of the ubiquitination process. The gene product of *cue1* contains the so-called CUE domain, which is conserved in Ascomycota and exhibits weak ubiquitin-binding properties. Proteins containing this domain are reported to participate in intramolecular monoubiquitylation [41]. The gene *ubi1* encodes a ubiquitin-activating enzyme (E1) with the subunit UBA2 and is involved in ubiquitination and subsequent protein processing [42]. The gene *sas3* encodes a histone acetyltransferase with a conserved MYST domain and is involved in the activation of gene transcription [43]. Information on those genes and their respective CV values can be found in Table 3.

**Figure 2:**
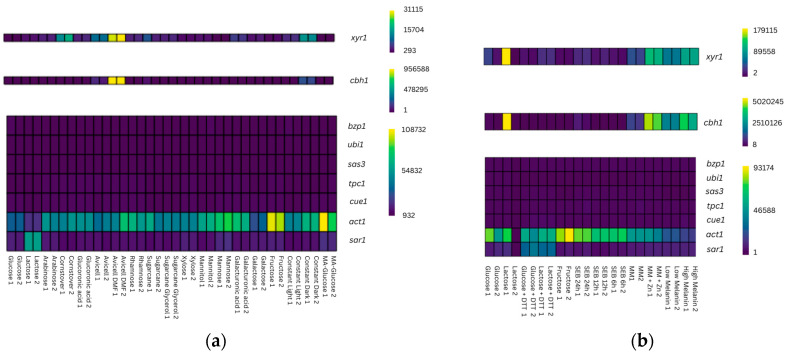
Expression heatmaps. Normalized counts of the investigated reference genes and the two well-characterized genes, *cbh1* and *xyr1*, obtained from the available WTS datasets of *T. reesei* strains QM6a (**a**) and Rut-C30 (**b**) in different growth conditions. The color gradient of the heatmap from purple (low normalized counts) and green (medium normalized counts) to yellow (high normalized counts) visualizes the strength of gene expression. MA, Mandels–Andreotti medium; DMF, dimethylformamide; DTT, dithiothreitol; SEB, sugarcane exploded bagasse, MM, minimal medium; 1 and 2 indicate biological duplicates. Based on the metadata provided with the publicly available WTS datasets, biological duplicates were included for each cultivation condition.

*Act1* and *sar1* are regulated in both *T. reesei* strains, QM6a and Rut-C30, depending on the cultivation condition according to available WTS data. Figure 2 provides the expression heatmaps of the five candidate reference genes, along with *act1*, *sar1*, and the genes *cbh1* and *xyr1* for QM6a and Rut-C30. The latter genes were used as control genes since their expression is well studied [44,45,46] and they are expected to give differences in expression in the used datasets. Figure 2 clearly illustrates that *act1* has a differential expression across many conditions, while *sar1* varies in some. In contrast, the new candidate reference genes maintain consistent expression levels across all conditions of the datasets.

### 3.2. Evaluation of Gene Expression Stability Using RT-qPCR and RefFinder

**Figure 3:**
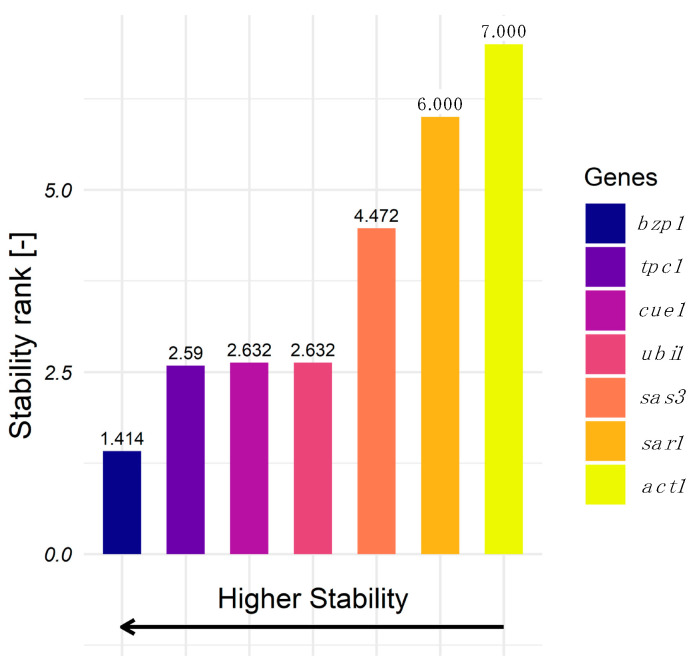
The stability ranking of the reference candidate genes and *act1* and *sar1* was calculated with RefFinder for all conditions. The reference genes are color-coded. Comprehensive ranking values were calculated based on Ct values obtained from qPCR, using biological duplicates and technical duplicates for each condition.

The applicability of the five identified candidate reference genes for gene expression analysis was assessed by RT-qPCR. For this purpose, a sample set was created by using two *T. reesei* strains (QM6a and Rut-C30), a range of different carbon sources, cultivation stages, and stress conditions. The Ct values obtained were analyzed with RefFinder [25], a tool that combines four algorithms for analyzing expression stability, resulting in a stability ranking. Figure 3 shows the gene expression stability ranking of the candidate reference genes and of the commonly used genes for normalization, *act1* and *sar1*. Out of the seven tested genes, *bzp1*, followed by *tpc1*, had the lowest variability and, thus, the most stable transcript levels.

**Figure 4:**
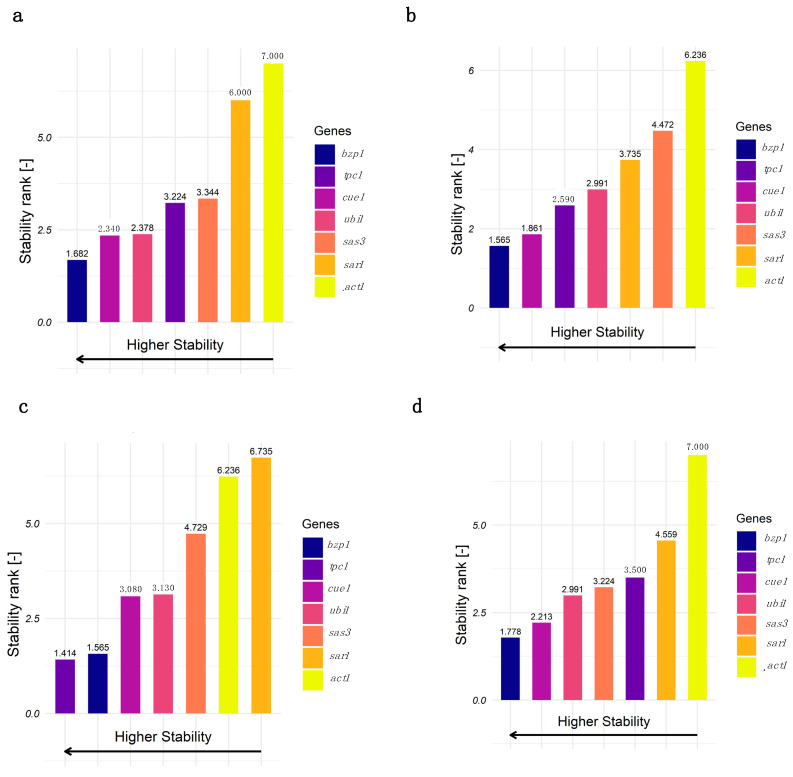
The stability ranking of the reference candidate genes and *act1* and *sar1* calculated with RefFinder separately for QM6a (**a**), Rut-C30 (**b**), early (**c**), and late (**d**) cultivation time points. The reference genes are color-coded. Comprehensive ranking values were calculated based on Ct values obtained from qPCR, using biological duplicates and technical duplicates for each condition.

The gene expression stability ranking was found to be overall consistent with slight changes when the data were split into different subgroups. Figure 4 shows the stability rankings separately for samples from QM6a and Rut-C30, and early and late cultivation time points. Only minor strain-specific changes in the gene expression stability rankings could be found. *Bzp1* and *cue1* are the two most stable genes in both QM6a and Rut-C30, and the other tested genes showed only slight differences in their ranking comparing the two strains. However, there are some interesting differences depending on the cultivation stage. In early cultivation stages, *tpc1* is the most and *sar1* the least stable gene, whereas in later cultivation stages, *bzp1* and *cue1* are the most stable genes, and *tpc1* is the third least stable. Despite these differences, *bzp1* is always ranked in first or second place, and *act1* and *sar1*, apart from one exception, in the last two.

### 3.3. Use of bzp1 and tpc1 as Reference Genes for Analyzing Gene Expression Using RT-qPCR

The RT-qPCR evaluation with RefFinder showed that all five selected genes are more stable than *act1* and *sar1*. Therefore, all of them could potentially serve as new reference genes. To exemplarily demonstrate their suitability for gene expression normalization, we selected *bzp1* and *tpc1* as they ranked as the two best genes in the all-condition stability ranking. Their performance was assessed by normalizing the expression of the well-characterized *cbh1* gene and comparing the results to the normalization with the commonly used reference genes *act1* and *sar1*.

**Figure 5:**
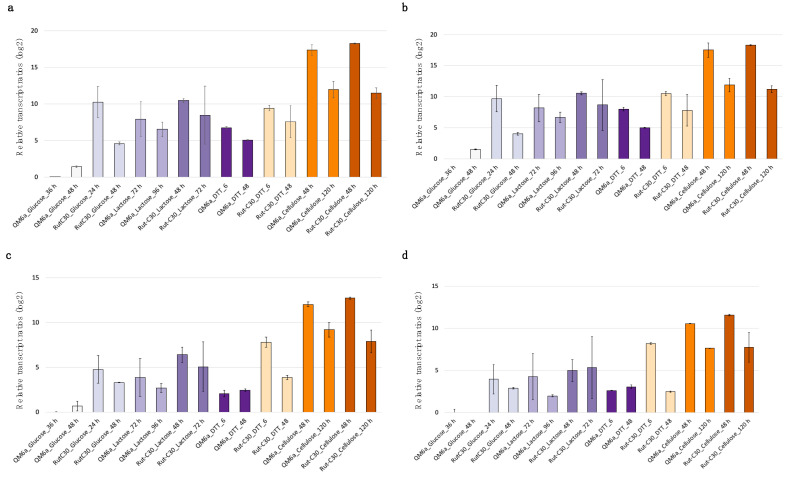
Relative transcript levels of *cbh1* using different genes for normalization. The *T. reesei* strains QM6a and Rut-C30 were cultivated in different cultivation conditions (glucose, lactose, lactose + DTT, cellulose), represented in different colors, and samples for RT-qPCR were taken at two different time points per condition. Relative *cbh1* transcript levels are given in log2-fold change normalized with the genes *bzp1* (**a**) or *tpc1* (**b**) or *sar1* (**c**) or *act1* (**d**). QM6a on glucose after 36 h was used as the reference condition. Mean values of biological and technical duplicates are provided and error bars indicate the standard deviation.

**Figure 6:**
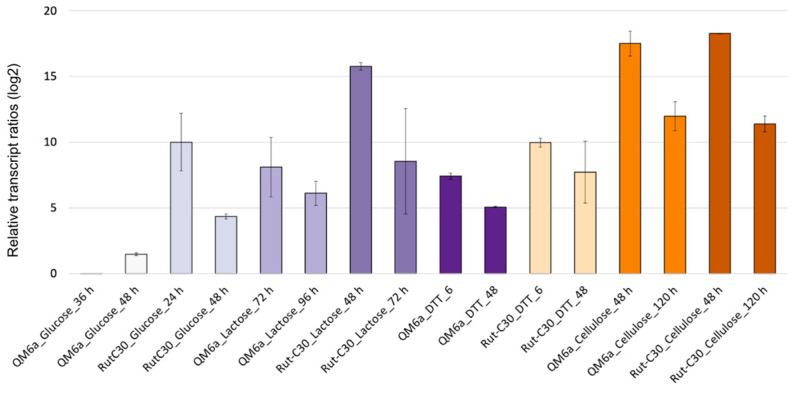
Relative transcript levels of *cbh1* using both *bzp1* and *tpc1* for normalization. The *T. reesei* strains QM6a and Rut-C30 were cultivated in different cultivation conditions (glucose, lactose, lactose + DTT, cellulose), represented in different colors, and samples for RT-qPCR were taken at two different time points per condition. Relative *cbh1* transcript levels are given in log2-fold change normalized with the genes *bzp1* and *tpc1*. QM6a on glucose after 36 h was used as the reference condition. Mean values of biological and technical duplicates are provided and error bars indicate the standard deviation.

For QM6a, *cbh1* expression is expected to be repressed by glucose, moderately induced by lactose, and strongly induced by cellulose [47,48]. In Rut-C30, a similar pattern is anticipated with the difference in a partial de-repression under glucose [26,49]. Under DTT-induced stress, *cbh1* mRNA levels are expected to decrease [50]. Figure 5 presents the *cbh1* transcript levels, normalized with both the new candidate reference genes (*bzp1* and *tpc1*) and the traditional ones (*act1* and *sar1*), using QM6a cultivated in glucose for 36 h as the reference condition. The overall expression pattern is similar, using *bzp1* or *tpc1* genes for normalization, and a similar pattern is also obtained by using *act1* or *sar1*. However, differences can be seen when comparing the pattern yielded using *bzp1* or *tpc1* compared to the pattern yielded by *act1* or *sar1*. For example, when ER stress is induced using DTT, differences arise. In QM6a at an earlier cultivation stage with DTT, *cbh1* expression appears higher when normalized with *bzp1* or *tpc1*, whereas normalization with *act1* and *sar1* suggests no significant change. In contrast, in Rut-C30, *cbh1* expression remains similar across time points when normalized with *bzp1* and *tpc1*, but when normalized with *act1* and *sar1*, it appears lower at the later stage compared to the beginning of cultivation. Also, the relative transcript ratios for all conditions are lower after normalization with *act1* and *sar1* compared to *bzp1* and *tpc1* (Figure 5). The combined use of *bzp1* and *tpc1* for normalization yields a highly similar result (Figure 6) compared to normalization with *bzp1* or *tpc1* alone (Figure 5a,b).

**Figure 7:**
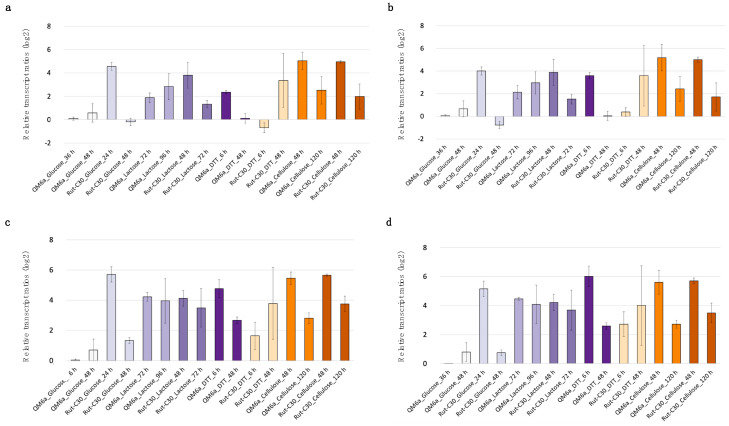
Relative transcript levels of *act1* and *sar1* using *bzp1* or *tpc1* for normalization. The *T. reesei* strains QM6a and Rut-C30 were cultivated in different cultivation conditions (glucose, lactose, lactose + DTT, cellulose), represented in various colors, and samples for RT-qPCR were taken at two different time points per condition. Relative transcript levels of *act1* (**a**,**b**) are given in log2-fold change normalized with the newly identified reference genes *bzp1* (**a**) or *tpc1* (**b**), and of *sar1* (**c**,**d**) with *bzp1* (**c**) or *tpc1* (**d**). QM6a on glucose after 36 h was used as the reference condition. Mean values of biological and technical duplicates are provided, and error bars indicate the standard deviation.

To finally clarify the potential regulation of the commonly used reference genes *act1* and *sar1*, their transcript levels were analyzed analogously using the *bzp1* and *tpc1* genes for normalization. The transcript patterns provided in Figure 7 reveal that both *act1* and *sar1* display differential expression depending on the strain and cultivation conditions. For example, in QM6a, *act1* and *sar1* show higher expression after 6 h in lactose with DTT compared to 48 h, while the opposite can be found in Rut-C30. This suggests that *act1* and *sar1* may be subject to strain-specific regulation in response to ER stress. This variation in regulation could explain the discrepancies observed in *cbh1* transcript normalization when using *act1* and *sar1* compared to the more stably expressed *bzp1* and *tpc1*. Generally, *act1* expression shows a more pronounced variation, aligning with the earlier findings from the WTS data analysis. Most importantly, the obtained *act1* and *sar1* expression patterns remain widely the same regardless of whether *bzp1* or *tpc1* was used as the gene for normalization, making them both ideal candidates as reference genes.

## 4. Discussion

Reference genes are essential for a reliable transcript analysis via RT-qPCR. The transcript abundance of a good reference gene represents the amount of isolated RNA and reversed transcribed cDNA and accounts for differences in RNA extraction and cDNA transcription efficiency. Using genes with fluctuating transcript levels causes an inaccurate interpretation of the expression of the target gene [51]. In the past, reference genes were selected mainly based on reports from the literature, followed by a characterization of their expression stability in a limited number of samples. For *T. reesei*, the genes *act1* and *sar1* were selected in this way and are, until now, commonly used for normalization. In this study, we identified novel reference gene candidates and characterized the expression stability of *act1* and *sar1* using publicly available WTS datasets from *T. reesei* strains QM6a and Rut-C30.

The commonly used genes *act1* and *sar1* are not universally stable, and therefore, they are not suitable as reference genes for every condition. This finding is in accordance with other reports from the literature showing that conventionally used reference genes differ in mRNA expression depending on cultivation conditions [52,53]. Several times *act1* was found to be differentially expressed in various conditions and, therefore, revealed to be unreliable as an internal reference for gene expression studies [24,51,54,55]. Actin was shown to be differentially expressed during growth or in response to biochemical stimuli, stress, and disease states [12,56]. In *Trichoderma atroviride*, both *act1* and *sar1* were observed to be less stable than other reference genes tested [52]. A study in 2015 tried to track the best reference genes for all filamentous fungi and found that actin is differentially regulated but identified *sarA* genes as promising candidates [57].

We would recommend validating the choice of reference genes for every experimental setup as it directly influences the study results. For example, we found that there seems to be a strain-specific regulation of *act1* and *sar1* in response to ER stress. Using *act1* and *sar1* for normalization would lead to an underestimation of *cbh1* transcript levels in the case of QM6a. Using regulated genes for normalization would cause a misinterpretation of target gene transcript abundance in these conditions and result in wrongfully drawn conclusions. The implications of misinterpreted expression data were also found in other studies [52,58,59,60]. For example, the use of *sar1* for normalization resulted in an overestimation of transcript levels of *pks4* (polyketide synthase) and *lox1* (lipoxygenase encoding gene) in *T. atroviride* [52]. Further, we identified some minor cultivation time and strain-specific effects on the stability of the reference genes we evaluated. Therefore, we want to emphasize that a reference gene must be rigorously tested for stability before being used in a new organism, strain, or experimental setup.

A key challenge in gene expression studies is the selection of new, reliable reference genes for accurate normalization. The approach in this study was to analyze public WTS data to identify five genes with high expression stability across two strains of *T. reesei* under various cultivation conditions. We confirmed a highly similar ranking of these genes regarding expression stability by RT-qPCR. The genes *bzp1* and *tpc1* were identified as the most stable, outperformed *act1* and *sar1*, and yielded reliable results when used for normalization. This underlines the accuracy of the chosen approach and is in accordance with other studies using WTS data to identify new reference genes [24,61].

Also, in other important fungal species, traditional reference genes are increasingly replaced by genes that were identified by omics-driven approaches. In *Aspergillus*, WTS analyses have identified, ubiquitin- and proteasome-related genes, amongst other candidates, as stable references. However, these were not experimentally validated [62]. In *F. graminearum*, a transcription factor was identified as one of the most stable genes using WTS data and found to be more stable than the traditionally used GAPDH [63]. That mirrors the identification of *bzp1* (transcription factor), *tpc1* (protein transport), and *cue1*/*ubi1* (ubiquitination) as stable candidates in *T. reesei* and emphasizes the potential of these genes for reliable normalization.

The consistent expression of *tpc1*, *cue1*, *ubi1*, and *sas3* is not unexpected, given their fundamental roles in cellular processes, and they fit into the type of commonly chosen reference genes. *Sas3* encodes a histone acetyltransferase and is involved in gene transcription regulation, which is continuously required for cellular functionality [43]. *Tpc1* is involved in protein trafficking, which is critical for maintaining cellular homeostasis under different conditions. To support cell viability and adaptation, its expression must be consistently maintained across different environmental and physiological states [40]. This central role in cellular logistics may explain the stable expression of *tpc1* and make it a promising candidate for a reference gene. Similarly, *cue1* and *ubi1* are part of the ubiquitination pathway, participating in protein regulation and degradation, which is equally vital for cellular adaptation and maintenance [41,42]. Their involvement in conserved mechanisms suggests their expression must remain relatively stable to ensure correct protein turnover.

Interestingly, *bzp1* was the most stably expressed gene, even though it could potentially be a transcriptional factor [38]. At first glance, using transcription factors as reference genes could be debatable, given their potential regulation of gene expression in specific conditions. On the other hand, it is not surprising to find a transcription factor having a stable transcript level because its regulation likely will not be on the transcriptional level. The abundance and the activity of a transcription factor can be controlled at various stages, from transcription to translation and posttranslational modifications [64,65]. In particular, the regulation at a late stage allows a transcription factor to exert its function fast after the receipt of a certain signal. One main reason why transcription factors initially might not have been commonly used as reference genes is that they are often expressed at low levels [66]. However, with PCR-based techniques, this is not a limitation anymore. The consistent expression observed within our tested conditions suggests that external factors do not strongly influence the gene expression of *bzp1*. Therefore, it is considered reliable for RT-qPCR normalization in *T. reesei*.

*Tpc1* and *bzp1* are likely conserved, as homologs were identified in other filamentous fungi within the pezizomycotina, including *A. niger*, *T. harzianum*, *T. aggressivum*, and *Aureobasidium pullulans*. In addition, *bzp1* orthologs were found also in *F. oxysporum*, *Penicillium flavigenum*, and other *Trichoderma* species. The presence of these orthologs further supports that *tpc1* and *bzp1* fulfill essential cellular roles and suggests that they could potentially be used as reference genes in other species as well.

Further validation is required to confirm the robustness of *bzp1* and *tpc1* as universal reference genes and their applicability in other related species. In general, it is unlikely that a universally applicable reference gene for all organisms and conditions exists. As discussed above, gene expression can differ between species depending on environmental conditions. So, even if *bzp1* and *tpc1* may not serve as universal reference genes, their reliable expression within defined conditions makes them valuable normalization candidates.

In essence, we consider both *bzp1* and *tpc1* to be suitable references for relative transcript analyses in *T. reesei* and suggest using them in combination, as recommended by the MIQE guidelines [13]. Additionally, we discourage using *act1* and *sar1* in *T. reesei* and always recommend validating reference genes in a new experimental setup.

## Figures and Tables

**Table 1:** Growth conditions and sampling time points for cultivations with *T. reesei* strains QM6a and Rut-C30.

Growth Condition	Cultivation Volume (mL)	QM6a	Rut-C30
Glucose	200	36 h/84 h	24 h/84 h
Lactose	200	72 h/96 h	48 h/72 h
Xylan	200	24 h/48 h	24 h/48 h
Glycerin	200	24 h/84 h	36 h/84 h
Cellulose	200	48 h/120 h	48 h/120 h
Lactose-DTT	50	6 h/48 h	6 h/48 h
Glucose-NaCl	50	6 h/24 h	6 h/24 h

**Table 2:** Primer sequences and amplification properties of genes used for the (RT)-qPCR.

Gene Name	Primer Sequences (5′-3′)	Amplicon Length (bp)
*act1*	Fwd: TGAGAGCGGTGGTATCCACG Rev: GGTACCACCAGACATGACAATGTTG	103
*sar1*	Fwd: TGGATCGTCAACTGGTTCTACGA Rev: GCATGTGTAGCAACGTGGTCTTT	115
*bzp1*	Fwd: GGCCTTTCTTTGAGCAGTGATG Rev: AGCTGCCCTTTGTTGTTGTC	92
*tpc1*	Fwd: TATGCGAATGAGCCGATTCC Rev: AACGTCCAGCTTCACATTGG	78
*cue1*	Fwd: GCGTAATCAAGGCGGTTCTG Rev: TGTTTTGCGGCTCGTTCTTG	108
*ubi1*	Fwd: TCAAATGCGGGCGACAAAAG Rev: TGTTGACCGGATGTTTGCAC	112
*sas3*	Fwd: ATCGCGTGCTGTACATTTGC Rev: TGTTTCGCAGCGCATTTGAG	91
*cbh1*	Fwd: ACTATGTCCAGAATGGCGTC Rev: TGGCGTAGTAATCATCCC	209

**Table 3:** Description of the investigated reference genes and their CV values in two *T. reesei* strains.

Gene Name	Gene Description	Transcript ID	CV (QM6a)	CV (Rut-C30)
*bzp1*	B-ZIP domain protein	TRIREDRAFT_50536	0.1450	0.1038
*tpc1*	Trafficking protein particle complex subunit 1	TRIREDRAFT_49838	0.2169	0.0993
*cue1*	CUE domain-containing protein	TRIREDRAFT_29932	0.2743	0.1691
*ubi1*	ubiquitin-like 1-activating enzyme E1 B	TRIREDRAFT_61945	0.1367	0.1340
*sas3*	Histone acetyltransferase SAS3	TRIREDRAFT_5916	0.1787	0.1237
*act1*	Actin	TRIREDRAFT_44504	0.3725	0.3534
*sar1*	Secretion-associated Ras-related GTPase	TRIREDRAFT_61470	0.9250	0.6846

## Data Availability

The public datasets used for this study can be found through the following links: https://www.ncbi.nlm.nih.gov/bioproject/PRJNA1097855 (accessed on 14 May 2025), https://www.ncbi.nlm.nih.gov/bioproject/PRJNA350272 (accessed on 14 May 2025), https://www.ncbi.nlm.nih.gov/bioproject/PRJNA695932 (accessed on 14 May 2025), https://www.ncbi.nlm.nih.gov/bioproject/PRJNA528215 (accessed on 14 May 2025), https://www.ncbi.nlm.nih.gov/bioproject/PRJNA510366 (accessed on 14 May 2025), https://www.ncbi.nlm.nih.gov/bioproject/PRJNA488233 (accessed on 14 May 2025), https://www.ncbi.nlm.nih.gov/bioproject/PRJNA392276 (accessed on 14 May 2025), https://www.ncbi.nlm.nih.gov/bioproject/PRJNA526091 (accessed on 14 May 2025), https://www.ncbi.nlm.nih.gov/bioproject/PRJNA948159 (accessed on 14 May 2025), https://www.ncbi.nlm.nih.gov/bioproject/PRJNA923496 (accessed on 14 May 2025), https://www.ncbi.nlm.nih.gov/bioproject/PRJNA977675 (accessed on 14 May 2025).

## Abbreviations

The following abbreviations are used in this manuscript:

DTT	Dithiothreitol
RT-qPCR	Reverse transcription–quantitative polymerase chain reaction
MA	Mandels–Andreotti
DMF	Dimethylformamide
SEB	Sugarcane exploded bagasse
MM	Minimal medium
CV	Coefficient of variation
MIQE	Minimum information for publication of quantitative real-time PCR experiments
WTS	Whole transcriptome sequencing
Ct	Threshold cycle
SRA	Sequence Read Archive
BCM	Basic culture medium

## Appendix A

### Appendix A.1

**Table A1:** WTS datasets of *T. reesei* QM6a.

Run	SRA ^1^ Study	Sequenced Bases	File Size	Platform	Condition	Reference
SRR23952272	SRP429031	7.6 G	2.1 Gb	Illumina NovaSeq 6000 (Illumina, San Diego, CA, USA)	glucose R1 ^2^	[67]
SRR23952273	SRP429031	6.8 G	1.9 Gb	Illumina NovaSeq 6000	glucose R2	
SRR8698740	SRP187914	7 G	2.9 Gb	Illumina HiSeq 2000 (Illumina, San Diego, CA, USA)	2% lactose (transfer after 12 h) R1	[68]
SRR8698741	SRP187914	7.8 G	3.2 Gb	Illumina HiSeq 2000	2% lactose (transfer after 12 h) R2	
SRR5765024	SRP110683	2.9 G	2.0 Gb	Illumina HiSeq 2500 (Illumina, San Diego, CA, USA)	1% sugarcane bagasse 48 h R1	[69]
SRR5765025	SRP110683	3.0 G	2.0 Gb	Illumina HiSeq 2500	1% sugarcane bagasse 48 h R2	
SRR5765030	SRP110683	3.2 G	2.1 Gb	Illumina HiSeq 2500	1% sugarcane bagasse (transfer from 1% glycerol) R1	
SRR5765031	SRP110683	4.0 G	2.7 Gb	Illumina HiSeq 2500	1% sugarcane bagasse (transfer from 1% glycerol) R2	
SRR7761289	SRP159003	2.8 G	1.8 Gb	Illumina HiSeq 2000	MA with 1% cellulose in constant light R1	[70]
SRR7761290	SRP159003	2.7 G	1.7 Gb	Illumina HiSeq 2000	MA with 1% cellulose in constant light R2	
SRR7761293	SRP159003	2.7 G	1.7 Gb	Illumina HiSeq 2000	MA with 1% cellulose in constant dark R1	
SRR7761294	SRP159003	2.6 G	1.6 Gb	Illumina HiSeq 2000	MA with 1% cellulose in constant dark R2	
SRR8329346	SRP173612	7.5 G	2.7 Gb	Illumina HiSeq X Ten (Illumina, San Diego, CA, USA)	MM with 1% Avicell 48 h R1	[71]
SRR8329347	SRP173612	6.9 G	2.4 Gb	Illumina HiSeq X Ten	MM with 1% Avicell 48 h R2	
SRR8329344	SRP173612	7.6 G	2.7 Gb	Illumina HiSeq X Ten	MM with 1% Avicell + 1% DMF 48 h R1	
SRR8329345	SRP173612	7.1 G	2.5 Gb	Illumina HiSeq X Ten	MM with 1% Avicell + 1% DMF 48 h R2	
SRR8756161	SRP188940	882.3 M	368.0 Mb	Illumina NextSeq 500 (Illumina, San Diego, CA, USA)	24 h on MA with glucose (transfer for 3 h to 1% D-Mannitol) R1	[72]
SRR8756162	SRP188940	738.7 M	306.6 Mb	Illumina NextSeq 500	24 h on MA with glucose (transfer for 3 h to 1% D-Mannitol) R2	
SRR25252694	SRP448956	2.7 G	908.6 Mb	Illumina NovaSeq 6000	MA with 1% corn stover (4 h transfer) R1	[73]
SRR25252695	SRP448955	2.7 G	908.0 Mb	Illumina NovaSeq 6000	MA with 1% corn stover (4 h transfer) R2	
SRR19551435	SRP378722	5.9 G	1.7 Gb	Illumina NovaSeq 6000	MA with 25 mM D-glucuronic acid (4 h transfer) R1	
SRR19551437	SRP378720	4.5 G	1.3 Gb	Illumina NovaSeq 6000	MA with 25 mM D-glucuronic acid (4 h transfer) R2	
SRR19551421	SRP378737	6.7 G	2.0 Gb	Illumina NovaSeq 6000	MA with 25 mM L-arabinose (4 h transfer) R1	
SRR19551434	SRP378723	6.9 G	2.1 Gb	Illumina NovaSeq 6000	MA with 25 mM L-arabinose (4 h transfer) R2	
SRR19551432	SRP378725	5.3 G	1.6 Gb	Illumina NovaSeq 6000	MA with 25 mM L-rhamnose (4 h transfer) R1	
SRR19551433	SRP378724	4.9 G	1.5 Gb	Illumina NovaSeq 6000	MA with 25 mM L-rhamnose (4 h transfer) R2	
SRR19551429	SRP378728	6.8 G	2.0 Gb	Illumina NovaSeq 6000	MA with 25 mM D-galacturonic acid (4 h transfer) R1	
SRR19551431	SRP378726	6.9 G	2.0 Gb	Illumina NovaSeq 6000	MA with 25 mM D-galacturonic acid (4 h transfer) R2	
SRR19551420	SRP378736	6.1 G	1.8 Gb	Illumina NovaSeq 6000	MA with 25 mM D-xylose (4 h transfer) R1	
SRR19551428	SRP378729	7.4 G	2.2 Gb	Illumina NovaSeq 6000	MA with 25 mM D-xylose (4 h transfer) R2	
SRR19551424	SRP378733	5.1 G	1.5 Gb	Illumina NovaSeq 6000	MA with 25 mM D-mannose (4 h transfer) R1	
SRR19551425	SRP378732	3.9 G	1.1 Gb	Illumina NovaSeq 6000	MA with 25 mM D-mannose (4 h transfer) R2	
SRR19551413	SRP378744	4.5 G	1.2 Gb	Illumina NovaSeq 6000	MA with 25 mM D-galactose (4 h transfer) R1	
SRR19551423	SRP378734	4.8 G	1.4 Gb	Illumina NovaSeq 6000	MA with 25 mM D-galactose (4 h transfer) R2	
SRR19551416	SRP378741	4.2 G	1.2 Gb	Illumina NovaSeq 6000	MA with 25 mM D-fructose (4 h transfer) R1	
SRR19551418	SRP378739	4.2 G	1.2 Gb	Illumina NovaSeq 6000	MA with 25 mM D-fructose (4 h transfer) R2	
SRR19551414	SRP378743	5.2 G	1.5 Gb	Illumina NovaSeq 6000	MA with 25 mM D-glucose (4 h transfer) R1	
SRR19551415	SRP378742	6.6 G	1.8 Gb	Illumina NovaSeq 6000	MA with 25 mM D-glucose (4 h transfer) R2	

^1^ SRA, Sequence Read Archive; ^2^ R, replicate.

**Table A2:** WTS datasets of *T. reesei* Rut-C30.

Run	SRA ^1^ Study	Sequenced Bases	File Size	Platform	Condition	Reference
SRR24768099	SRP440257	6.3 G	2.0 Gb	Illumina NextSeq 2000 (Illumina, San Diego, CA, USA)	Fed batch with lactose + 10 mM DTT for 2 h R1 ^2^	[74]
SRR24768104	SRP440257	5.9 G	1.9 Gb	Illumina NextSeq 2000	Fed batch with lactose + 10 mM DTT for 2 h R2	
SRR24768100	SRP440257	4.6 G	1.4 Gb	Illumina NextSeq 2000	Fed batch with lactose R1	
SRR24768103	SRP440257	5.2 G	1.6 Gb	Illumina NextSeq 2000	Fed batch with lactose R2	
SRR24768101	SRP440257	5.3 G	1.6 Gb	Illumina NextSeq 2000	Fed batch with glucose + 10 mM DTT for 2 h R1	
SRR24768102	SRP440257	5.7 G	1.7 Gb	Illumina NextSeq 2000	Fed batch with glucose + 10 mM DTT for 2 h R2	
SRR24768105	SRP440257	4.7 G	1.4 Gb	Illumina NextSeq 2000	Fed batch with glucose R1	
SRR24768106	SRP440257	4.5 G	1.3 Gb	Illumina NextSeq 2000	Fed batch with glucose R2	
SRR4446960	SRP091982	2.4 G	1.6 Gb	Illumina HiSeq 2000 (Illumina, San Diego, CA, USA)	BCM with 1% fructose 48 h + 24 h R1	[75]
SRR4446961	SRP091982	2.5 G	1.7 Gb	Illumina HiSeq 2000	BCM with 1% fructose 48 h + 24 h R2	
SRR4446958	SRP091982	2.5 G	1.7 Gb	Illumina HiSeq 2000	BCM with 1% fructose 48 h (transfer to 0.5% SEB 24) R1	
SRR4446959	SRP091982	2.5 G	1.7 Gb	Illumina HiSeq 2000	BCM with 1% fructose 48 h (transfer to 0.5% SEB 24 h) R2	
SRR4446955	SRP091982	2.3 G	1.6 Gb	Illumina HiSeq 2000	BCM with 1% fructose 48 h (transfer to 0.5% SEB 12 h) R1	
SRR4446956	SRP091982	2.7 G	1.8 Gb	Illumina HiSeq 2000	BCM with 1% fructose 48 h (transfer to 0.5% SEB 12 h) R2	
SRR4446953	SRP091982	2.5 G	1.7 Gb	Illumina HiSeq 2000	BCM with 1% fructose 48 h (transfer to 0.5% SEB 6 h) R1	
SRR4446954	SRP091982	2.4 G	1.7 Gb	Illumina HiSeq 2000	BCM with 1% fructose 48 h (transfer to 0.5% SEB 6 h) R2	
SRR23088649	SRP417642	6.6 G	1.9 Gb	Illumina NovaSeq 6000	1% Avicell 48 h R1	[76]
SRR23088650	SRP417642	7.2 G	2.1 Gb	Illumina NovaSeq 6000	1% Avicell 48 h R2	
SRR23088646	SRP417642	8.3 G	2.4 Gb	Illumina NovaSeq 6000	1% Avicell 48 h + 3 mM Zn2+ R1	
SRR23088647	SRP417642	7.1 G	2.0 Gb	Illumina NovaSeq 6000	1% Avicell 48 h + 3 mM Zn2+ R2	
SRR28595945	SRP500486	7.9 G	2.4 Gb	Illumina HiSeq 4000 (Illumina, San Diego, CA, USA)	high-melanin necromass from *Hyaloscypha bicolor* R1	[77]
SRR28595946	SRP500486	8.5 G	2.6 Gb	Illumina HiSeq 4000	high-melanin necromass from *H. bicolor* R2	
SRR28595948	SRP500486	8.0 G	2.5 Gb	Illumina HiSeq 4000	low-melanin necromass from *H. bicolor* R1	
SRR28595949	SRP500486	8.5 G	2.6 Gb	Illumina HiSeq 4000	low-melanin necromass from *H. bicolor* R2	

^1^ SRA, Sequence Read Archive; ^2^ R, replicate.

## Appendix B

### Appendix B.1

The following section contains exemplary commands used in the Terminal of Debian 12 that were used to extract count information from the WTS files.

Indexing of genomes for further use in HISAT2:
  hisat2-build genome.fa genome

Writing a SAM file from paired-end RNA-seq files:
  hisat2 -x genome -1 sample_R1.fastq.gz -2 sample_R2.fastq.gz -S output.sam

Writing a SAM file from single-end RNA-seq file:
  hisat2 -x genome_index -U sample.fastq.gz -S output.sam

Conversion from SAM to BAM format via SAMtools:
  samtools sort -l 9 -o output.bam -@ 4 output.sam

Extraction of raw counts from the SAM file:
  featureCounts -p -t exon -g gene_id -a annotation.gtf -o output.txt
  output.bam

The following code was used in R to receive normalized counts from raw counts with DeSeq2. This code should serve as an example: it is reduced to only two conditions in duplicates, all output folder information is not displayed, and it was adapted for every condition.

If (!requireNamespace(“BiocManager”, quietly = TRUE)) {
  install.packages(“BiocManager”)
  }
  BiocManager::install(“DESeq2”)
  BiocManager::install(“EnhancedVolcano”)
  install.packages(“writexl”)
  library(writexl)
  library(DESeq2)
  library(EnhancedVolcano)

cond1_1 <- read.table(“C:/---/cond1_1.txt”, header = TRUE, sep =
  “\t”, stringsAsFactors = FALSE)
  cond1_2 <- read.table(“C:/---/cond1_2.txt”, header = TRUE, sep =
  “\t”, stringsAsFactors = FALSE)
  cond2_1 <- read.table(“C:/---/cond2_1.txt”, header = TRUE, sep =
  “\t”, stringsAsFactors = FALSE)
  cond2_2 <- read.table(“C:/---/cond2_2.txt”, header = TRUE, sep =
  “\t”, stringsAsFactors = FALSE)

countData1_cond1_1 <- cond1_1[, 7:ncol(cond1_2)]
  countData1_cond1_2 <- cond1_2[, 7:ncol(cond1_2)]
  countData2_cond2_1 <- cond2_1[, 7:ncol(cond2_1)]
  countData2_cond2_2 <- cond2_1[, 7:ncol(cond2_2)]

countData1_cond1_1 <- as.data.frame(countData1_cond1_1)
  countData1_cond1_2 <- as.data.frame(countData1_cond1_2)
  countData2_cond2_1 <- as.data.frame(countData2_cond2_1)
  countData2_cond2_2 <- as.data.frame(countData2_cond2_2)

rownames(countData1_cond1_1) <- cond1_1$Geneid
  rownames(countData1_cond1_2) <- cond1_2$Geneid
  rownames(countData2_cond2_1) <- cond2_1$Geneid
  rownames(countData2_cond2_2) <- cond2_2$Geneid

countData<-cbind(countData1_cond1_1,countData1_cond1_2,
  countData2_cond2_1, countData2_cond2_2)
  colnames(countData) <- c(“Condition1 1”, “Condition1 2”)

ncol(countData)

sampleInfo <- data.frame(
  row.names = colnames(countData),
  condition = factor(rep(c(“Condition1”, “Condition2”), each = 2)),
  replicate = factor(rep(c(“Rep1”, “Rep2”), 2))
  )

dds <- DESeqDataSetFromMatrix(countData = countData, colData =
  sampleInfo, design = ~ condition)
  dds <- estimateSizeFactors(dds)
  normalized_counts <- counts(dds, normalized=TRUE)
  normalized_counts_df <- as.data.frame(normalized_counts)
  normalized_counts_df$GeneID <- rownames(normalized_counts_df)
  normalized_counts_df <- normalized_counts_df[,
  c(ncol(normalized_counts_df), 1:(ncol(normalized_counts_df– - 1))]

write_xlsx(normalized_counts_df“ “C:/---/normalized_counts.xl”x”)

### Appendix B.2

**Table A3:** Ct values (averages of technical duplicates) of RT-qPCR analysis for candidate reference genes, *act1* and *sar1*, and cultivation conditions of QM6a and Rut-C30 in biological duplicates.

Strain	C-Source	Time Point	Replicate	Ct Values							
				*sar1*	*act1*	*bzp1*	*tpc1*	*cue1*	*ubi1*	*sas3*	*cbh1*
QM6a	Cellulose	120	1	19.80	19.56	18.50	19.55	18.83	19.48	20.59	17.00
QM6a	Cellulose	120	2	21.45	21.80	18.51	19.60	19.83	19.81	21.29	18.45
QM6a	Cellulose	48	1	18.46	17.35	19.26	20.74	19.57	20.59	22.09	11.00
QM6a	Cellulose	48	2	18.78	18.04	18.59	19.79	18.91	19.83	21.01	11.48
QM6a	DTT	6	1	19.84	22.19	19.97	22.40	19.60	21.08	21.66	24.65
QM6a	DTT	6	2	20.51	21.92	19.60	21.75	19.69	19.97	21.04	24.55
QM6a	DTT	48	1	21.38	23.29	18.35	19.05	18.97	18.51	19.37	26.00
QM6a	DTT	48	2	21.90	24.03	18.51	19.34	19.66	18.63	19.77	26.55
QM6a	Glucose	36	1	23.96	22.87	17.18	18.04	17.38	17.36	18.40	30.50
QM6a	Glucose	36	2	23.95	22.68	17.27	17.97	17.45	17.42	18.45	30.60
QM6a	Glucose	84	1	23.44	22.54	18.41	19.24	18.89	19.10	20.35	30.65
QM6a	Glucose	84	2	24.87	24.10	18.58	19.61	19.75	19.62	21.09	31.00
QM6a	Glycerin	84	1	23.83	24.88	21.37	21.98	22.19	20.77	22.01	n.m. ^1^
QM6a	Glycerin	84	2	24.10	25.60	20.07	20.96	21.28	19.59	21.09	n.m.
QM6a	Glycerin	24	1	17.08	16.81	18.72	19.52	17.93	19.26	20.89	n.m.
QM6a	Glycerin	24	2	17.26	16.08	19.02	19.69	17.97	19.26	20.87	n.m.
QM6a	Lactose	96	2	20.81	20.13	19.03	20.06	19.99	18.63	19.68	19.70
QM6a	Lactose	96	1	21.55	20.33	17.26	18.30	18.19	16.47	17.73	24.05
QM6a	Lactose	72	1	19.10	21.37	17.93	18.77	18.82	17.88	18.98	23.75
QM6a	Lactose	72	2	19.67	20.73	17.88	19.15	19.25	18.20	19.37	23.40
QM6a	NaCl	6	1	21.08	21.01	18.55	19.80	19.12	18.87	19.71	n.m.
QM6a	NaCl	6	2	19.90	19.99	18.22	19.49	18.72	18.67	19.68	n.m.
QM6a	NaCl	24	1	19.16	19.24	17.88	19.13	18.06	19.02	20.22	n.m.
QM6a	NaCl	24	2	19.77	20.30	18.88	20.46	19.20	20.13	21.48	n.m.
QM6a	Xylan	48	1	21.62	22.38	18.82	20.26	20.54	19.18	20.65	n.m.
QM6a	Xylan	48	2	21.08	21.80	21.31	23.02	22.52	21.77	23.26	n.m.
QM6a	Xylan	24	1	18.89	18.75	17.93	18.98	18.65	18.13	19.39	n.m.
QM6a	Xylan	24	2	18.12	18.03	19.02	19.95	19.11	19.41	20.37	n.m.
Rut-C30	Cellulose	120	1	20.66	20.73	18.80	19.76	19.23	19.65	20.87	9.90
Rut-C30	Cellulose	120	2	20.32	21.33	17.61	18.34	17.84	18.24	19.26	9.90
Rut-C30	Cellulose	48	1	17.77	17.36	18.60	19.68	18.88	19.85	21.13	19.25
Rut-C30	Cellulose	48	2	17.69	17.36	18.38	19.40	18.61	19.46	20.56	16.90
Rut-C30	DTT	6	1	23.88	22.19	19.67	21.70	21.20	20.22	21.43	20.65
Rut-C30	DTT	6	2	25.49	25.82	19.11	21.24	20.32	18.49	19.45	20.85
Rut-C30	DTT	48	1	20.53	19.97	20.81	22.24	20.11	21.38	22.54	23.45
Rut-C30	DTT	48	2	20.96	20.73	16.79	17.86	17.63	17.31	18.56	23.45
Rut-C30	Glucose	84	1	23.04	23.98	18.54	19.39	19.08	19.24	20.59	21.75
Rut-C30	Glucose	84	2	23.84	24.92	18.76	19.82	19.93	19.20	20.50	19.10
Rut-C30	Glucose	24	1	18.62	18.82	18.53	19.33	18.91	19.37	20.43	26.50
Rut-C30	Glucose	24	2	18.58	18.39	19.56	20.64	20.07	20.54	22.19	26.55
Rut-C30	Glycerin	36	1	20.50	19.42	17.84	18.60	18.13	17.10	17.99	n.m.
Rut-C30	Glycerin	36	2	21.53	20.69	17.11	18.17	18.00	16.78	17.80	n.m.
Rut-C30	Glycerin	84	1	18.87	20.41	18.36	19.58	19.02	19.29	20.40	n.m.
Rut-C30	Glycerin	84	2	18.02	19.29	18.41	19.50	19.17	18.78	20.07	n.m.
Rut-C30	Lactose	48	1	19.96	20.20	21.54	22.46	21.24	21.55	23.05	16.40
Rut-C30	Lactose	48	2	19.68	20.86	20.21	21.25	21.03	20.33	21.88	14.40
Rut-C30	Lactose	72	1	21.74	22.29	18.18	19.11	19.37	18.23	19.39	25.20
Rut-C30	Lactose	72	2	20.01	22.33	18.75	20.07	19.98	18.87	20.17	18.70
Rut-C30	NaCl	6	1	20.38	19.90	17.73	19.20	18.14	18.77	19.73	n.m.
Rut-C30	NaCl	6	2	19.95	19.28	17.31	18.60	17.53	18.09	18.92	n.m.
Rut-C30	NaCl	24	1	20.13	19.27	18.10	19.34	18.44	18.56	19.35	n.m.
Rut-C30	NaCl	24	2	21.28	20.47	18.29	18.94	18.74	18.26	19.11	n.m.
Rut-C30	Xylan	48	1	21.95	21.70	17.57	18.67	18.59	17.65	18.80	n.m.
Rut-C30	Xylan	48	2	21.50	21.31	22.20	24.18	22.84	21.93	23.46	n.m.
Rut-C30	Xylan	24	1	20.05	18.94	20.10	21.32	20.61	19.67	21.04	n.m.
Rut-C30	Xylan	24	2	19.82	19.01	19.50	20.59	20.14	19.16	20.34	n.m.

^1^ n.m., not measured.

**Table A4:** Individual rankings and values for DeltaCT, BestKeeper, Normfinder, and Genorm calculated with RefFinder for subgroups QM6a, Rut-C30, and early and late time points.

Subgroup	Calculation Method	Gene	Rank	Calculated Value ^1^
QM6a	DeltaCT	*bzp1*	1	1.310
		*tpc1*	3	1.336
		*cue1*	2	1.330
		*ubi1*	4	1.374
		*sas3*	5	1.396
		*act1*	7	2.211
		*sar1*	6	2.033
QM6a	BestKeeper	*bzp1*	1	0.92
		*tpc1*	4	1.01
		*cue1*	3	0.99
		*ubi1*	2	0.95
		*sas3*	5	1.10
		*act1*	7	1.91
		*sar1*	6	1.64
QM6a	Normfinder	*bzp1*	2	0.702
		*tpc1*	3	0.840
		*cue1*	1	0.547
		*ubi1*	4	0.959
		*sas3*	5	0.995
		*act1*	7	2.017
		*sar1*	6	1.753
QM6a	Genorm	*ubi1*/*sas3*	1/2	0.508
		*tpc1*	3	0.717
		*bzp1*	4	0.778
		*cue1*	5	0.865
		*sar1*	6	1.314
		*act1*	7	1.570
Rut-C30	DeltaCT	*bzp1*	1	1.213
		*cue1*	2	1.226
		*tpc1*	3	1.257
		*ubi1*	4	1.359
		*sas3*	5	1.457
		*act1*	6	2.037
		*sar1*	7	2.038
Rut-C30	BestKeeper	*ubi1*	1	1.04
		*cue1*	2	1.17
		*bzp1*	3	1.20
		*sas3*	4	1.20
		*tpc1*	5	1.35
		*sar1*	6	1.48
		*act1*	7	1.56
Rut-C30	Normfinder	*cue1*	1	0.527
		*bzp1*	2	0.568
		*tpc1*	3	0.725
		*ubi1*	4	0.854
		*sas3*	5	1.139
		*act1*	6	1.823
		*sar1*	7	1.826
Rut-C30	Genorm	*bzp1*/*tpc1*	1/2	0.448
		*cue1*	3	0.541
		*sas3*	4	0.764
		*ubi1*	5	0.823
		*act1*	6	1.303
		*sar1*	7	1.513
Early	DeltaCT	*Tpc1*	1	1.279
		*Bzp1*	2	1.300
		*cue1*	3	1.359
		*ubi1*	4	1.462
		*sas3*	5	1.582
		*act1*	6	2.125
		*sar1*	7	2.190
Early	BestKeeper	*bzp1*	1	0.93
		*ubi1*	2	0.94
		*cue1*	3	1.02
		*tpc1*	4	1.06
		*sas3*	5	1.19
		*sar1*	6	1.43
		*act1*	7	1.58
Early	Normfinder	*tpc1*	1	0.503
		*cue1*	2	0.572
		*bzp1*	3	0.637
		*ubi1*	4	1.023
		*sas3*	5	1.300
		*act1*	6	1.882
		*sar1*	7	1.987
Early	Genorm	*bzp1*/*tpc1*	1/2	0.609
		*ubi1*	3	0.791
		*sas3*	4	0.837
		*cue1*	5	0.903
		*act1*	6	1.383
		*sar1*	7	1.614
Late	DeltaCT	*bzp1*	1	1.120
		*cue1*	2	1.126
		*sas3*	3	1.164
		*Ubi1*	4	1.181
		*tpc1*	5	1.236
		*sar1*	6	1.698
		*act1*	7	1.841
Late	BestKeeper	*Ubi1*	1	1.00
		*sar1*	2	1.19
		*sas3*	3	1.19
		*cue1*	4	1.22
		*bzp1*	5	1.23
		*tpc1*	6	1.30
		*act1*	7	1.52
Late	Normfinder	*cue1*	1	0.555
		*bzp1*	2	0.607
		*sas3*	3	0.722
		*Ubi1*	4	0.737
		*tpc1*	5	0.926
		*sar1*	6	1.451
		*act1*	7	1.663
Late	Genorm	*bzp1*/*tpc1*	1/2	0.574
		*cue1*	3	0.638
		*sas3*	4	0.766
		*ubi1*	5	0.785
		*sar1*	6	1.138
		*act1*	7	1.339

^1^ DeltaCT and BestKeeper Standard deviation, Normfinder, and Genorm-stability value.
